# One-Step Fast Fabrication of Electrospun Fiber Membranes for Efficient Particulate Matter Removal

**DOI:** 10.3390/polym16020209

**Published:** 2024-01-11

**Authors:** Huanliang Liu, Wenqing Lai, Yue Shi, Lei Tian, Kang Li, Liping Bian, Zhuge Xi, Bencheng Lin

**Affiliations:** 1 Tianjin Institute of Environmental and Operational Medicine, Tianjin 300050, China; tjliuhuanliang@126.com (H.L.); laiwenqing0316@126.com (W.L.); october2144@163.com (Y.S.); tjtianlei@126.com (L.T.); tjlikang@126.com (K.L.); bmoglp@126.com (L.B.); 2 Tianjin Key Laboratory of Risk Assessment and Control Technology for Environment & Food Safety, Tianjin 300050, China

**Keywords:** electrospinning, air filtration, polysulfone, PM_2.5_, PM_1.0_

## Abstract

Rapid social and industrial development has resulted in an increasing demand for fossil fuel energy, which increases particulate matter (PM) pollution. In this study, we employed a simple one-step electrospinning technique to fabricate polysulfone (PSF) fiber membranes for PM filtration. A 0.3 g/mL polymer solution with an *N*,*N*-dimethylformamide:tetrahydrofuran volume ratio of 3:1 yielded uniform and bead-free PSF fibers with a diameter of approximately 1.17 μm. The PSF fiber membrane exhibited excellent hydrophobicity and mechanical properties, including a tensile strength of 1.14 MPa and an elongation at break of 116.6%. Finally, the PM filtration performance of the PSF fiber membrane was evaluated. The filtration efficiencies of the membrane for PM_2.5_ and PM_1.0_ were approximately 99.6% and 99.2%, respectively. The pressure drops were 65.0 and 65.2 Pa, which were significantly lower than those of commercial air filters. Using this technique, PSF fiber membrane filters can be easily fabricated over a large area, which is promising for numerous air filtration systems.

## 1. Introduction

With the development of human society, air pollution, particularly particulate matter (PM), has significantly increased, causing serious health problems [1]. PM includes solid and liquid particles in air. It can be categorized as PM_10_, PM_2.5_, and PM_1.0_ based on the particle size [2,3,4]. Among them, PM_2.5_ and PM_1.0_ are the main constituents of airborne PM. Because of their diminutive sizes, they can easily permeate into human lung tissue and circulatory systems upon inhalation, causing heart and respiratory tract disorders [5,6]. To date, filtration is the most popular and effective method of removing PM from the air. Thus, there is an urgent need for simple methods for fabricating high-performance air filters.

Fiber filters have attracted significant attention for addressing PM pollution because of their high interception efficiency, favorable air permeability, and scalability [7,8]. Fiber filters employ four mechanisms to filter PM: interception, inertial impact, Brownian diffusion, and electrostatic effect [9]. Currently, meltblown polypropylene (PP) nonwoven fabric is commonly used in air filters [10]. Commercial filters are fabricated via meltblowing. To produce meltblown polypropylene (PP) fabrics, PP granules and electret masterbatches are blended and meltblown into PP fibers, which are then electrotreated via corona charging. According to Patra et al. [11], the filtration efficiencies of surgical masks and N95 masks, commercially available filters, for PM with particle sizes of 0.3–10 µm are 77.8% and 91.8%, respectively. Stahl et al. [12] also reported that the filtration efficiencies of N95 and N99 masks for PM with a particle size of approximately 0.3 µm are 54.1% and 90.5%, respectively. Because of their relatively large fiber diameter, these commercial filters cannot efficiently filter small particles in the air [13]. In addition, their fabrication processes are complex and expensive. Moreover, PP filters are associated with the rapid depletion of electrostatic charges, which reduces their PM filtration efficiency [14]. Recently, various polymeric fibers, including polyacrylonitrile (PAN) [15], polyvinyl alcohol (PVA) [16], polyvinylidene chloride (PVC) [17], and polyvinylidene fluoride (PVDF) [18], have been explored for PM filtration. Zhang et al. [19] synthesized PVA-based nanofiber membranes using a spinning method and fabricated a novel fiber filter using a thermal crosslinking technique. The PM_2.5_ removal efficiency of the fiber membrane was above 95%, and the pressure drop was less than 100 Pa. Liu et al. [20] combined spinning and spraying techniques to fabricate a PVA/ethyl cellulose composite membrane, which recorded filtration efficiencies of 99.74% and 99.77% for PM_1.0_ and PM_2.5_, respectively. Yao et al. [21] prepared monodispersed ZnO particles using template- and surfactant-free hydrothermal methods and fabricated PVA-co-PE fibers using a melt extrusion phase-separation technique. The air filter was obtained by coating a mixture of ZnO particles and a PVA-co-PE fiber suspension onto a PP meltblown nonwoven fabric, and the filtration efficiency for sodium chloride aerosol with a particle size of 1.0 μm was more than 99.9%. Zheng et al. [22] synthesized self-supporting three-dimensional (3D) PVDF nanofiber membranes with crimped patterns and an optimum filtration efficiency of 93.6% for PM. Zhang et al. [23] fabricated a novel polyvinyl alcohol (PVA)/cellulose nanocrystal (CNC) composite nanofiber filter via electrowinning and reported a PM_2.5_ filtration efficiency of 99.1%. Despite their excellent filtration performance, these filters are limited by their complex preparation processes, the high cost of raw materials, and poor scalability. Therefore, there is an urgent need to develop new filter materials that can be fabricated via simple and low-cost process, which will promote their large-scale production and diverse applications.

Polysulfone (PSF) is a thermoplastic polymer with remarkable chemical stability and excellent mechanical and thermal properties, which are attributed to its unique molecular structure [24]. A PSF molecular unit consists of a sulfonyl group, an ether group, and isopropylidene groups in the main chain [25]. Because of its special molecular structure and excellent physicochemical properties, PSF is widely used in water and blood filtration. Li et al. [26] synthesized a PSF/rGO/ZnO composite ultrafiltration membrane using a simple and green method, and the membrane showed good performance in dye wastewater treatment. Zhong et al. [27] fabricated a PSF-based hemodialysis membrane with excellent hemocompatibility and biocompatibility, and the membrane exhibited excellent hemodialysis performance superior to that of conventional membranes. However, to date, PSF has not been explored for air filtration.

Compared with other fiber fabrication techniques, including self-assembly [28], the template method [29], and phase separation [30], electrospinning is simple and versatile [31]. In principle, electrospinning involves the use of high-voltage electrostatic force to generate a polymer melt or solution jet [32]. The jet initially moves in a straight path and gradually transitions into a spiral trajectory as it travels. Concurrently, the solvent volatilizes, and a fiber membrane is formed on the collector. Although considerable progress has been made in single-nozzle electrospinning, it can only fabricate small-fiber membranes. To fabricate large-scale fiber membranes, needleless electrospinning has emerged as a promising technique. Electrospun fiber membranes offer several advantages, including small and uniform diameters, high specific surface areas, interconnected pore structures, and good mechanical properties, making them promising for air filtration [33]. Moreover, in situ charge injection into fiber membranes can be achieved during electrospinning. The electrospinning process enables bulk charges to be captured in the membrane and generates dipole charges. Charges in the membrane improve the attraction of PM to the fibers, thus increasing the PM filtration efficiency [34,35]. 

**Figure 1:**
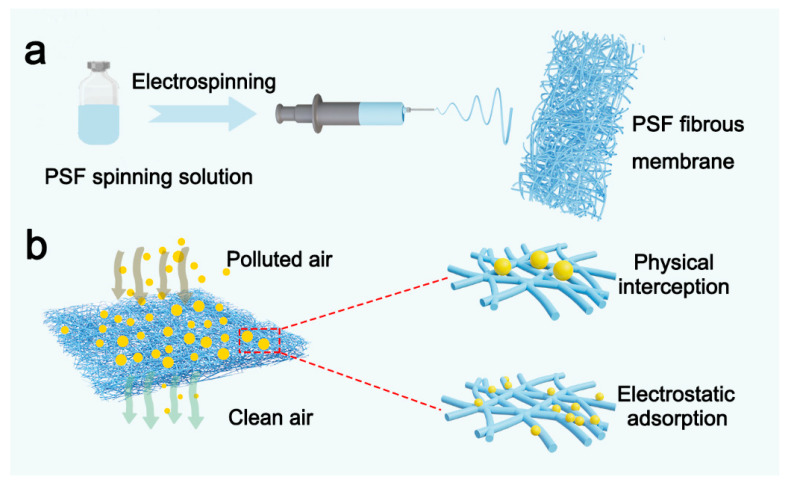
(**a**) Preparation process and (**b**) filtration mechanism of the polysulfone (PSF) fiber membranes.

In this study, we employed one-step electrospinning to fabricate PSF-based fiber filters and investigated their PM filtration performance. Figure 1 shows the synthesis process and filtration mechanisms of the filter membranes. We investigated the effect of PSF solution concentration and solvents on the PSF fiber synthesis. Then, we fabricated PSF fiber membranes with different thicknesses by adjusting the spinning time. Furthermore, the physical and chemical properties of PSF fiber membranes were comprehensively examined using scanning electron microscopy (SEM), Fourier-transform infrared spectroscopy (FTIR), and water contact angle (WCA) analysis. Finally, the PM filtration performance of the PSF fiber membranes and the underlying mechanism were investigated.

## 2. Materials and Methods

### 2.1. Materials

PSF (P-1800; Mw: 68,102 g/mol; *ρ*: 1.24 g/cm^3^) was purchased from Solvary Company (Long Beach, CA, USA). *N*,*N*-dimethylformamide (DMF) and tetrahydrofuran (THF) were purchased from Aladdin. All materials and solvents were used as received without further purification.

### 2.2. Fabrication of the PSF Fiber Membranes

PSF fiber membranes were fabricated using a homemade electrospinning system. Various masses of PSF (25, 30, and 35 wt%) were dissolved in 4 mL of solvent (DMF:THF volume ratio = 3:1) for 12 h. Subsequently, the solutions were placed on an electrospinning device, and fibers were formed on aluminum foil under a high-voltage power supply of 8 kV. The distance between the nozzle and the collector was 20 cm, and the flow rate was maintained at 0.8 mL/h. Ultrathin PSF fiber membranes with different thicknesses were obtained by varying the electrospinning time (60, 90, 120, and 150 min), and the corresponding samples were denoted as PSF-1, PSF-2, PSF-3, and PSF-4, respectively. The fabricated PSF fiber membranes were vacuum-dried at 50 °C for 24 h to eliminate the residual solvent.

### 2.3. Characterization

The morphology of the prepared PSF fibers was characterized using SEM (SU8010, Hitachi, Tokyo, Japan). To measure the average diameter of the PSF fibers, 100 fibers were randomly selected from the SEM images. Each fiber diameter was measured using ImageJ software (v1.8.0), and the average diameter was calculated. The average pore size of the fiber membrane was determined by randomly selecting 100 voids in the fibers from the SEM images. The diameters of the inner circles in the voids were obtained using ImageJ, and the average value was recorded as the pore size of the fiber membrane. The fiber membranes were further characterized using FTIR (FTIR, Nicolet iS10, Billerica, MA, USA). The surface wettability of the membranes was determined by WCA measurements (DSA25, Kruss, Hamburg, Germany). A 2 μL of water droplet was dropped at three locations on the sample surface, and the mean WCA was determined. An electrostatic tester (JH-TEST, Shenzhen, China) was used to measure the surface potential of the fiber membranes in situ. The probe was slowly moved close to the targeted measurement point until two LED light spots from the electrostatic tester completely overlapped, and the value was displayed. The mechanical properties of the fiber membranes, including tensile strength, elongation at break, and Young’s modulus, were measured using a tensile testing machine (Sunszy-2000, utm2103, Suns, Shenzhen, China). Furthermore, the porosity of the fiber membranes was determined using the volume replacement method. The samples were immersed in anhydrous ethanol for 5 min until saturation, and their masses after ethanol absorption were recorded. The porosity of the samples was then calculated using Equation (1).

(1) $$P=\frac{\left(M_{1}-M_{0}\right)}{\left(\rho \times V\right)}\times 100\%,$$

where *M*_0_ and *M*_1_ are the masses of the sample before and after ethanol absorption, respectively, *V* the volume of the sample, and *ρ* the density of ethanol. All samples were measured three times.

### 2.4. Filtration Performance of the PSF Fiber Membranes

The TSI 8532 automated filtration tester was used to evaluate the filtration performance of the PSF fiber membranes. The unit was connected to a particle generator that produced particles with diameters of 0.3–10.0 μm. A fan was connected to the particle generator to propel the generated aerosol particles through the filters. The aerosol wind speed was measured using an anemometer (FMA1002R-V1, OMEGA, New York, NY, USA). Two solid-state laser photometers were employed to measure the aerosol concentrations and particle size distributions before and after passing through the filters. The pressure drop across the filter was measured using a pressure transducer (DP-CLCTM5825, TSI, Missoula, MT, USA). Particulate aerosol tests were conducted at room temperature with a fixed aerosol wind speed of 0.25 km/h, and the filtration efficiency (η) of the filters was calculated using Equation (2):

(2) $$\eta =\frac{\xi 1-\xi 2}{\xi 1}\times 100\%,$$

where ξ1 and ξ2 are the particle concentrations upstream and downstream of the filter, respectively. The pressure drop ∆P (the difference in pressure before and after filtration) was calculated as follows:

(3) $$∆P=P_{2}-P_{1},$$

where *P*_1_ and *P*_2_ are the pressures before and after filtration, respectively.

The quality factor (*QF*) is a crucial parameter for evaluating the filtration performance of filters and can be calculated as follows:

(4) $$QF=\frac{-\ln\left(1-\eta \right)}{∆P},$$

To evaluate the durability of PSF-2, the surface electrostatic potential, filtration efficiency, pressure drop, and *QF* were evaluated for 28 days. The fiber membranes were stored at room temperature and 50% relative humidity, and the indices, including surface electrostatic potential, filtration efficiency, pressure drop, and *QF*, were tested every 7 days.

### 2.5. Statistical Analysis

We used Microsoft Excel 2021 to calculate the average values and standard deviations (SDs) of the indices. The obtained SD was used as the error bar, and the final results were expressed as mean ± SD.

## 3. Results and Discussion

### 3.1. Effects of Solution Concentration and Solvent Composition on the Spinnability of the PSF Solutions

We first investigated the effects of the polymer solution concentration and solvent composition on the morphology of the resulting fibers. Poor spinnability is indicated by the presence of beads in the spun fibers, and good spinnability is indicated by homogeneous but discontinuous fibers (some charged droplets deposit on the fiber membrane). Excellent spinnability is indicated by homogeneous and continuous fibers. The PSF solution with a concentration of 25 wt% showed poor spinnability (Table 1). Regardless of the solvent (pure DMF, pure THF, or a mixture of DMF and THF), PSF fibers with a beaded structure were obtained, as shown in Figure S1. Polymer solutions play a key role in electrostatic spinning. When the concentration of the polymer solution is low, the entanglement between the polymer chains is weak; thus, fibers with many beads are obtained [36,37]. Therefore, we increased the concentration of the polymer solution to 30 wt%. Pure THF was used as the solvent, and good spinnability was achieved, as indicated by homogeneous but discontinuous fibers. This is attributed to occasional nozzle clogging and the deposition of charged droplets on the fiber membranes, as shown in Figure S2. When a mixture of 3 mL of DMF and 1 mL of THF was used as the solvent, the PSF solution exhibited excellent spinnability, as indicated by the homogeneous and continuous fibers (Figure S3). Furthermore, we increased the polymer concentration to 35 wt%, but the spinning needles were completely blocked, preventing the formation of fibers. The optimal spinning conditions included a solution of 30 wt% and a mixed solvent containing 3 mL of DMF and 1 mL of THF.

### 3.2. Morphology of the PSF Fiber Membranes

Based on the optimal conditions, we further synthesized PSF fiber membranes with different thicknesses by varying the fiber deposition time from 60 to 150 min. The thicknesses of the four PSF fiber membranes obtained were 32, 60, 100, and 120 μm. We further characterized the PSF fiber membranes using FTIR spectroscopy (Figure S4). All PSF fiber membranes exhibited the same characteristic bands. The peaks at 1588 and 1242 cm^−1^ were attributed to the C=C and C–O–C stretching vibrations, respectively, and the peak at 1151 cm^−1^ was ascribed to the symmetrical O=S=O telescoping vibration.

**Figure 2:**
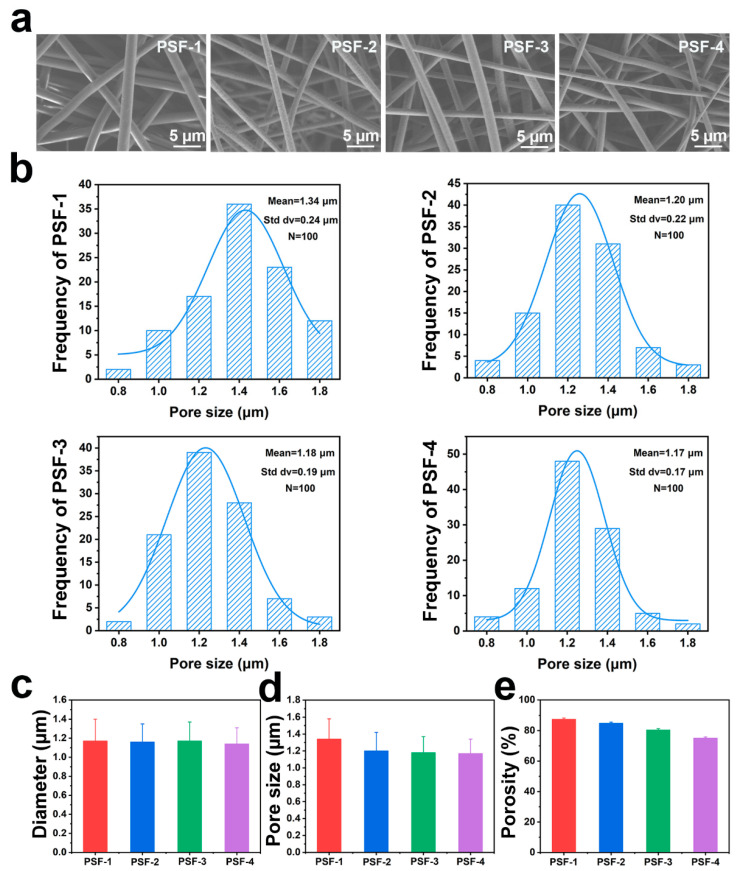
(**a**) SEM image, (**b**) pore-size distribution, (**c**) average fiber diameters, (**d**) average pore sizes, and (**e**) porosity of the PSF fiber membranes.

Figure 2 shows the microscopic morphology of the PSF fiber membranes. All fibers were uniform and bead-free (Figure 2a). The average fiber diameters in the four PSF fiber membranes were approximately 1.17 μm (Figure 2c). This is because the PSF fibers were fabricated using the same concentration and solution composition. The pore-size distribution was evaluated, and the average pore size of the PSF fiber membranes was calculated, as shown in Figure 2b,d. For the four samples, the pore size slightly decreased with increasing thickness (from 1.34 µm for PSF-1 to 1.17 µm for PSF-4). PSF-1, PSF-2, PSF-3, and PSF-4 showed porosities of 87.5%, 84.9%, 80.5%, and 75.1%, respectively (Figure 2e). These results indicate that the pore structures (pore size and porosity) of the fiber membranes can be tuned by varying their thickness. During the electrospinning process, fibers are deposited on the collector layer by layer, and as the thickness of the membrane increases, the fibers stack tightly, resulting in a gradual decrease in both the pore size and porosity [38]. The obtained porosity and pore size were comparable to those reported for electrospun fiber membranes. For example, Zhu and Cai reported a relatively small pore size of approximately 1 µm and high porosity (77.0–84.9%) for electrospun fiber membranes [39,40].

### 3.3. Mechanical Properties of the PSF Fiber Membranes

**Figure 3:**
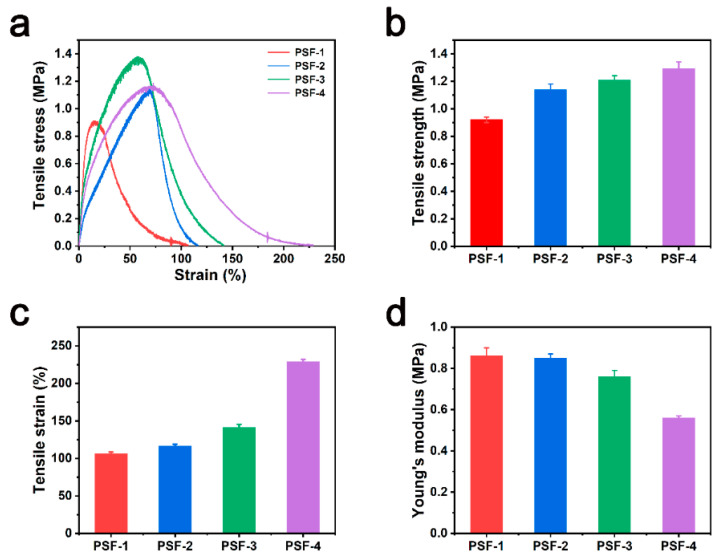
(**a**) Stress–strain curve, (**b**) tensile strength, (**c**) elongation at break, and (**d**) Young’s modulus of the PSF fiber membranes.

Figure 3 shows the mechanical properties of the synthesized PSF fiber membranes. Figure 3a shows the stress–strain curves. The tensile strength of PSF-1, PSF-2, PSF-3, and PSF-4 was 0.92, 1.14, 1.21, and 1.29 MPa (Figure 3b), and their elongation values at break were approximately 106.2%, 116.6%, 141.3%, and 229.0%, respectively (Figure 3c). As shown in Figure 3d, the Young’s modulus of the membranes slightly decreased with increasing thickness (0.86, 0.85, 0.76, and 0.56 MPa for PSF-1, PSF-2, PSF-3, and PSF-4, respectively). These results show that the mechanical properties of PSF membranes can be improved by tuning the thickness of the membrane. During the electrospinning process, the fibers stack more tightly as the deposition time increases. Friction between fibers increases with the thickness of the membrane, which makes thicker membranes more prone to fracture [41]. Zhu et al. [42] reported an electrospun fiber membrane with good mechanical properties, including a tensile strength of 0.8 MPa and an elongation at break of 16%. Zhang et al. [43] also reported an electrospun fiber membrane with a tensile strength of 0.89 MPa and an elongation at break of 8.99%. According to Zhang et al. [44], the tensile strength of disposable masks is 2.5 MPa. The mechanical properties of our PSF fiber membranes were comparable to those reported for fiber filters.

Electrospun PSF fibers have been reported in the literature [45,46]. These studies focused on some spinning parameters that influenced the fiber morphology; however, in this study, we investigated in detail the effects of spinning parameters, including polymer solution concentration, solvent composition, spinning voltage, and tip-to-collector distance, on the morphology of the fiber. In addition, previous studies have considered the morphology of single fibers, especially the roughness and diameter, whereas we considered the surface morphology of single fibers (fiber diameter and roughness) and the fiber aggregate structure, including pore size and porosity. Furthermore, we investigated the chemical composition, wetting behavior, and mechanical properties of the PSF fibers.

### 3.4. Filtration Performance of the PSF Fiber Membranes and the Underlying Mechanism

**Figure 4:**
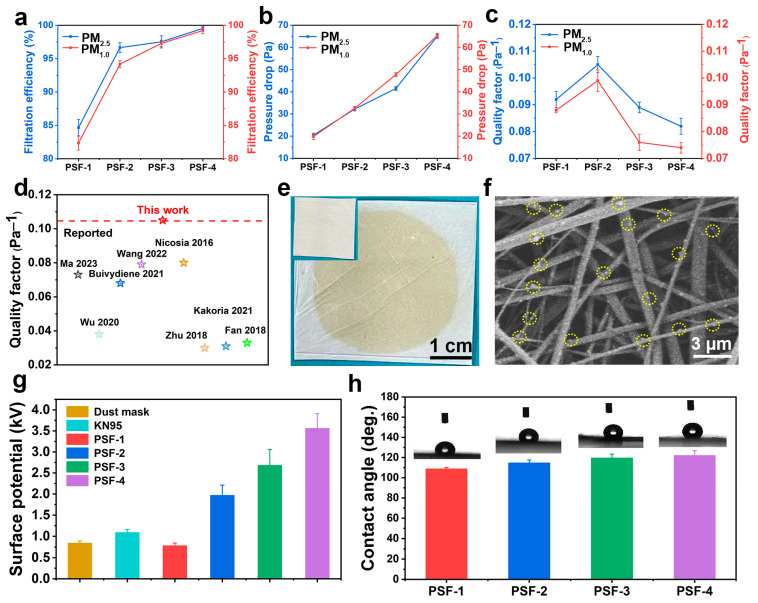
(**a**) Filtration efficiency, (**b**) pressure drop, and (**c**) *QF* of the PSF fiber membranes. (**d**) Comparison of the *QF*s of the PSF fiber membranes with those of the reported filters [16,38,50,51,52,53,54,55]. (**e**) Appearance and (**f**) SEM image of the PSF fiber membranes after PM_2.5_ filtration (the inset shows the appearance of the membranes before filtration). (**g**) Surface potential of the PSF fiber membranes. (**h**) WCAs of the PSF fiber membranes.

Figure 4 shows the filtration performance of the PSF fiber membranes with different thicknesses and the corresponding filtration mechanism. The filtration efficiency of the membranes for PM_2.5_ and PM_1.0_ increased with the thickness of the membrane. For PM_2.5_, PSF-1, PSF-2, PSF-3, and PSF-4 exhibited filtration efficiencies of 84.7%, 96.7%, 97.5%, and 99.6%, respectively, and for PM_1.0_, their efficiencies were 82.4%, 94.2%, 97.3%, and 99.2%, respectively. Previous studies have shown that electrospun fiber membranes exhibit good particle-trapping effects, making them promising for air filtration. Shao et al. [47] reported above 98.5% PM filtration efficiency for modified PLA nanofiber membranes. Zhang et al. [48] fabricated a novel dendritic cellulose nanofiber membrane with a PM filtration efficiency of 98.37%. Peng et al. [49] prepared a self-charging air filtration mask using the friction electric effect between electrospun poly(vinylidene fluoride) nanofiber film and nylon fabric. The membrane showed a filtration efficiency of 95.8% for 0.3 μm particles.

A drop in pressure is the change in static pressure as air moves through a filter. It reflects the respiratory resistance and breathing comfort of the filter [56,57]. Therefore, we studied the pressure drop in all PSF fiber membranes. For PM_2.5_, the pressure drop in the fiber membranes increased from 20.4 Pa for PSF-1 to 65.0 Pa for PSF-4 (Figure 4b). A similar trend was obtained for PM_1.0_ filtration. This is because, compared with a thin membrane, a thick membrane can easily intercept PM, thereby increasing the respiratory resistance of the membrane. Segovia et al. [58] demonstrated that the pressure drop of an ordinary mask for particles was approximately 200 Pa, whereas that of an N95 mask was as high as 600 Pa. These results show that PSF fiber filters exhibit lower respiratory resistance than both ordinary and N95 masks. To achieve a better balance between filtration efficiency and pressure drop, the *QF* values of the fiber membranes were calculated, as shown in Figure 4c. For PM_2.5_ filtration, the *QF* of the PSF-1 fiber membrane was 0.092 Pa^−1^, which was lower than that of PSF-2 (0.105 Pa^−1^). The *QF* values of the PSF-3 and PSF-4 fiber membranes (0.089 and 0.082 Pa^−1^, respectively) were lower than those of PSF-1 and PSF-2. A similar trend was observed for PM_1.0_ filtration (0.088, 0.099, 0.076, and 0.074 Pa^−1^ for PSF-1, PSF-2, PSF-3, and PSF-4, respectively). Owing to its high *QF* for PM_2.5_ and PM_1.0_, PSF-2 is considered the optimal membrane and was chosen for further study. We also compared the *QF* of the fiber membranes with that of the reported filters. The *QF* of the PSF fiber membranes was higher than that of the reported filters, indicating the good filtration performance and low respiratory resistance of our fiber membranes (Figure 4d) [16,38,50,51,52,53,54,55].

To understand the filtration mechanism of the PSF membranes, the macroscopic appearance and microscopic morphology of the PSF fiber membranes after PM_2.5_ filtration were observed. The PSF fiber membranes after filtration showed a distinct yellow area in the filtration region, indicating that a large amount of PM was intercepted by the PSF fiber membranes (Figure 4e). The SEM results further revealed that many small particulate matter particles were tightly absorbed on the outer surfaces of the PSF fibers (Figure 4f). Electrostatic charges involved in electrostatic adsorption by the PSF fiber membranes may play a vital role in PM interception; therefore, we investigated the variation of the surface electrostatic potential of the PSF fiber membranes via membrane thickness [59]. The surface electrostatic potential of two commercially available masks was also evaluated as a control (Figure 4g). The results showed that the surface electrostatic potential of a common dust mask and a KN95 filter was 0.84 and 1.09 kV, respectively, and that of PSF-1, PSF-2, PSF-3, and PSF-4 was 0.78, 1.97, 2.68, and 3.56 kV, respectively. This shows that the surface electrostatic potential of PSF-2, PSF-3, and PSF-4 was larger than that of the commercial filters, and thicker PSF membranes exhibited higher surface electrostatic potential.

PSF fiber formation during electrospinning is driven by electric field forces. A high voltage is generally exerted on the polymer solution. When the fibers form, electrical charges are simultaneously integrated into the fibers. When the spinning voltage is up to 8 kV, a high electrostatic charge remains in the fibers. Because of the large surface electrostatic potential, PSF fibers can highly absorb PM, resulting in excellent PM filtration efficiency of the membrane [60]. 

Previous studies have shown that the surface static charges of filters may quickly be lost in high-humidity environments, which affects the PM filtration of the filter [61]. In this study, the WCA values of the PSF-1, PSF-2, PSF-3, and PSF-4 fiber membranes were 108°, 114°, 119°, and 122°, respectively, indicating strong hydrophobic properties of the PSF membranes (Figure 4h). The hydrophobic properties of the PSF fiber membranes can be attributed to the chemical composition and rough surface structure of the fibers. PSF contains many hydrophobic groups, including phenyl and isopropyl [62]. After the electrospinning process, a rough fiber surface can be obtained, and air can be captured in the voids. Owing to the repellency effect of the trapped air, the PSF fiber membranes showed strong hydrophobic properties. The water resistance of the membrane prevents not only the dissipation of electrostatic charges from the PSF membrane but also the fouling of externally contaminated liquids [63,64].

Furthermore, the effects of high voltage and tip-to-collector distance on the porosity and PM filtration efficiency of the PSF fiber membranes were investigated (Figure S5). The results indicate that both high voltage and tip-to-collector distance influence the porosity and PM filtration efficiency of the PSF fiber membranes. As the voltage increased from 6 to 10 kV, the porosity of the PSF fiber membranes decreased from 86.0% to 83.8%, and the corresponding filtration efficiency increased from 95.2% to 96.9%. In addition, the PM filtration efficiency of the PSF fiber membranes decreased with increasing tip-to-collector distance. As the tip-to-collector distance increased from 15 to 25 cm, the porosity of the fiber membranes increased from 83.2% to 85.0%, and the PM filtration efficiency decreased from 97.2% to 95.6%. We further investigated the effects of the spinning voltage and tip-to-collector distance on the surface electrostatic potential (Figure S6). There was no significant change in the surface electrostatic potential of the fiber membranes as voltages and tip-to-collector distances varied. This further indicates that both high voltage and tip-to-collector distance affect the porosity and filtration efficiency of fiber membranes.

### 3.5. Comparison of the Filtration Performance of PSF-2 and Commercial Filters

**Figure 5:**
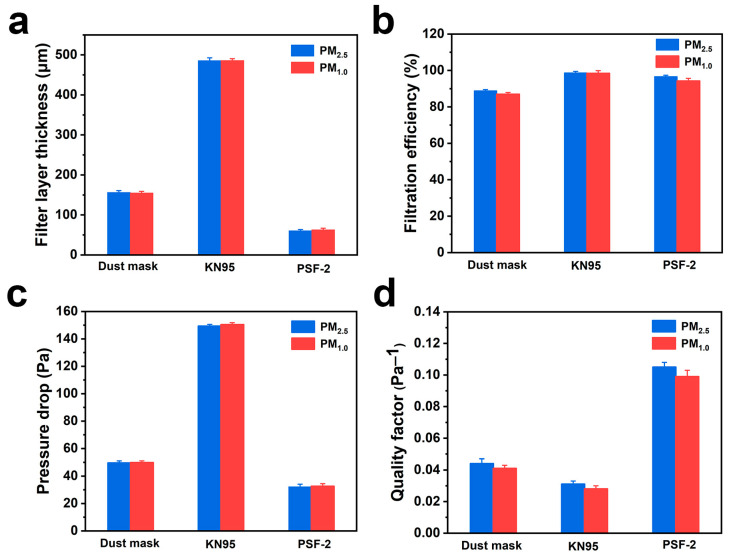
Comparison of the (**a**) thickness, (**b**) filtration efficiency, (**c**) pressure drop, and (**d**) *QF* of the PSF-2 fiber membrane with those of commercial filters.

Figure 5 compares the thickness, filtration efficiency, pressure drop, and *QF* of PSF-2, a common dust mask, and KN95 filters. As shown in Figure 5a, the filter layers of the common dust mask and KN95 filter had thicknesses of 156.2 and 485.4 µm, respectively, which were much higher than that of the PSF-2 fiber membrane (~60 µm). The filtration efficiencies of the dust mask and KN95 filter for PM_2.5_ were 88.8% and 98.7% (Figure 5b). The efficiency of the PSF-2 fiber membrane was higher than that of the dust mask and comparable to that of KN95. A similar trend was observed for PM_1.0_ filtration. Furthermore, the pressure drop of the dust mask and KN95 filter for PM_2.5_ filtration was 49.6 and 149.3 Pa, respectively, which was significantly higher than that of the PSF-2 fiber membrane (32.3 Pa) (Figure 5c). Alternatively, as shown in Figure 5d, the *QF* of the PSF-2 fiber membrane was much larger than that of the two commercial filters (0.105 and 0.099 Pa^−1^ for PM_2.5_ and PM_1.0_ filtration, respectively). According to the National Standard GB 2626-2019 of the People’s Republic of China [65], the filtration efficiency of a filter should be higher than 95% and the pressure drop should be less than 170 Pa, both of which were satisfied by the PSF-2 fiber membrane.

### 3.6. Filtration Durability of the PSF-2 Fiber Membrane

**Figure 6:**
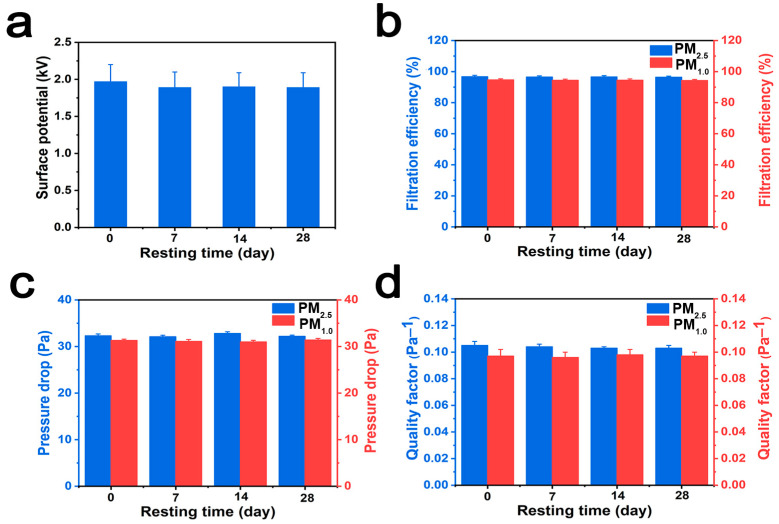
(**a**) Electrostatic dissipation, (**b**) filtration efficiency, (**c**) pressure drop, and (**d**) *QF* of the PSF-2 fiber membrane.

To evaluate the durability of the membrane, the surface electrostatic potential, filtration efficiency, pressure drop, and *QF* of the PSF-2 fiber membrane were assessed for 28 days. Figure 6a shows that the electrostatic potential on the PSF fiber membrane surface remained stable over 28 days. In addition, no significant changes were observed in the filtration efficiency (Figure 6b), pressure drop (Figure 6c), and *QF* (Figure 6d) of the fiber membrane after PM_2.5_ and PM_1.0_ filtration. These results indicate the long-term durability of the PSF fiber membrane for PM filtration.

Recent studies have shown that the electrostatic trapping of surface charges plays an important role in PM filtration. Qiu et al. [66] prepared a self-powered air filter based on a breath-driven friction nanogenerator with filtration efficiencies of 95% for PM_0.3_ and 86.3% after 2 h of use. Peng et al. [49] produced a respirator-inspired, self-charging air-filtering mask with a reduction filtration efficiency of 95.8% after 60 h of effective use. These show that the electrostatic charge generated by friction dissipates after use, thereby affecting the filtration efficiency. Zhang et al. [67] reported that corona charges are much more stable than frictional charges and exhibit long-term stability. This is because corona charges can transfer from a “shallow trap” to a “deep trap”. In this study, fibers were formed by applying high voltage to a polymer solution. During this process, charges were simultaneously injected into the fibers because of the high spinning voltage of 8 kV, resulting in a large amount of electrostatic charge remaining in the fibers. In addition, high electrospinning voltage promotes corona charge formation; thus, numerous stable corona charges can be injected into the fiber. Because the WCA of a fiber membrane is approximately 120°, it is not easily wetted, which promotes the storage of electrostatic charge. Therefore, the surface electrostatic potential of the PSF fiber filters did not change significantly after 28 days of use. Finally, the PSF fiber membrane showed long-term stability because of its large and continuous surface electrostatic potential.

## 4. Conclusions

In summary, PSF fiber membranes were fabricated using a simple one-step electrospinning method. The fiber membranes exhibited highly porous structures and good mechanical and hydrophobic properties. The porosity of the fiber membranes could be tuned by varying the spinning parameters (high voltage and tip-to-collector distance). Because of its low porosity and abundant injected charges, the PSF-2 fiber membrane exhibited exceptional filtration efficiency (99.6% and 99.2% for PM_2.5_ and PM_1.0_, respectively) and minimal pressure drop (65.0 and 65.2 Pa for PM_2.5_ and PM_1.0_, respectively), which were much higher than those of commercial dusk masks and KN95 respirators. Furthermore, no obvious electrostatic dissipation was observed over 28 days. The filtration efficiency, pressure drop, and *QF* of the PSF fiber membrane remained unchanged after 28 days, indicating the long-term durability of the fiber membrane during PM filtration. Because of the simplicity of the manufacturing procedures and the low cost of the raw materials, the PSF fiber membrane can be fabricated into various shapes and sizes and assembled into high-performance masks and air conditioning filters. The simple preparation process, low cost, and high PM removal efficiency make PSF fiber membranes promising for various industrial filtration applications.

## Figures and Tables

**Table 1:** The spinnability of various PSF solutions.

PSF Solution Concentration (wt%)	Solvent Composition (4 mL)	Spinnability
25 wt%	Pure THF	Poor
	Pure DMF	Poor
	DMF:THF = 3:1	Poor
30 wt%	Pure THF	Good
	Pure DMF	Poor
	DMF:THF = 3:1	Excellent
35 wt%	Pure THF	No
	Pure DMF	No
	DMF:THF = 3:1	No

## Data Availability

All required data are in the article.

## Supplementary Materials

The following supporting information can be downloaded at: https://www.mdpi.com/article/10.3390/polym16020209/s1.
