# Supplementary material for: One-Step Fast Fabrication of Electrospun Fiber Membranes for Efficient Particulate Matter Removal
Source: Polymers (Basel). 2024 Jan 11;16(2):209. doi: 10.3390/polym16020209 (PMC10818706; doi:10.3390/polym16020209)
Supplement: Supplementary file 1 [file polymers-16-00209-s001.zip › polymers-2755862-supplementary.pdf]

Supporting information

One-step fast fabrication of electrospun fiber membrane for efficient particular matter removal

Huanliang Liu [1,2,†], Wenqing Lai [1,2,†], Yue Shi [1,2], Lei Tian [1,2], Kang Li [1,2], Liping Bian [1,2], Zhuge Xi [1,2,*] and Bencheng Lin [1,2,*]

[1] Tianjin Institute of Environmental and Operational Medicine, Tianjin 300050, China; tjliuhuanliang@126.com (H.L.); laiwenqing0316@126.com (W.L.); october2144@163.com (Y.S.); tjtianlei@126.com (L.T.); tjlikang@126.com (K.L.); bmoglp@126.com (L.B.)

[2] Tianjin Key Laboratory of Risk Assessment and Control Technology for Environment & Food Safety, Tianjin 300050, China

* Correspondence: zhugexi2003@sina.com (Z.X.); linbencheng123@126.com (B.L.); Tel.: +86-022-84655124; +86-022-84655324

† These authors contributed equally to this work.

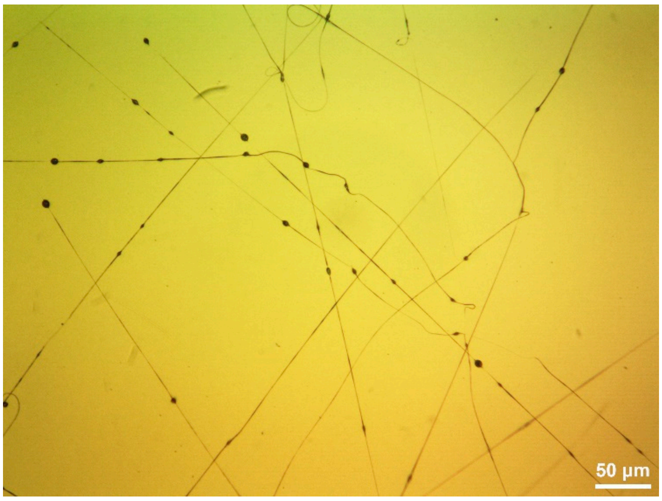

Figure S1. Bead-on-string structured PSF fibers (poor spinnability).

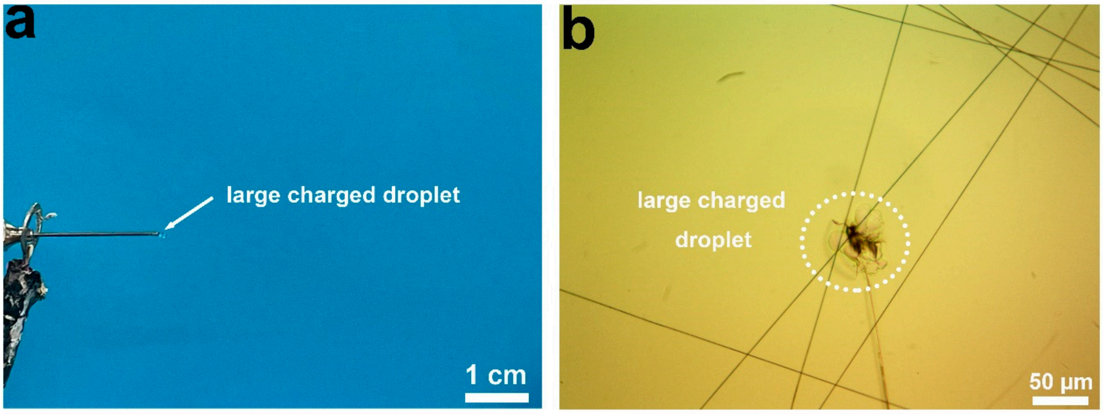

Figure S2. (a) Digital photograph of large charged droplet and (b) optical microscope picture of charged droplet on the PSF fibers (good spinnability).

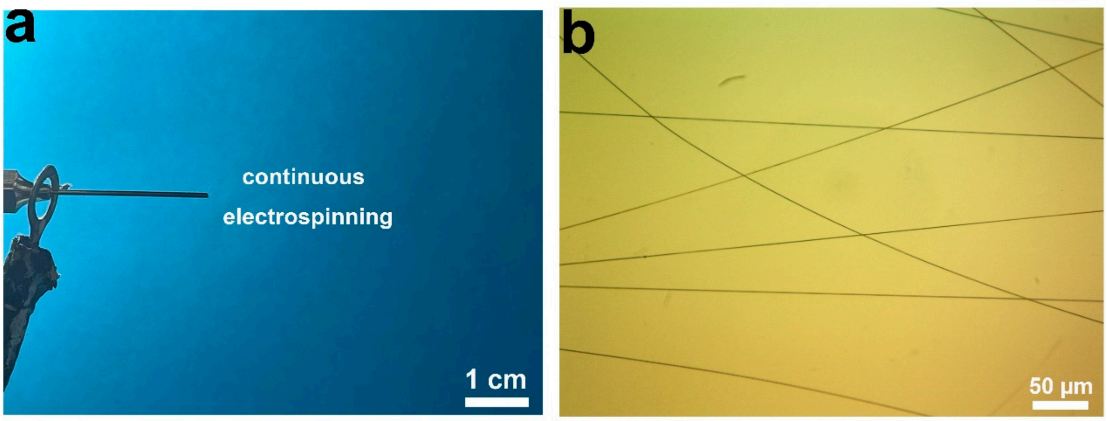

Figure S3. (a) Digital photograph of the electrospinning process and (b) optical microscope picture of the smooth PSF fibers (excellent spinnability).

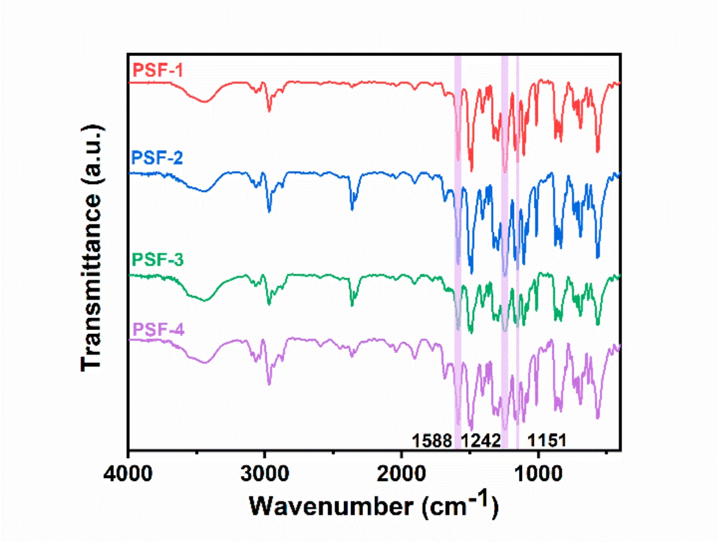

Figure S4. FTIR spectra of PSF-1, PSF-2, PSF-3, PSF-4 fiber membranes.

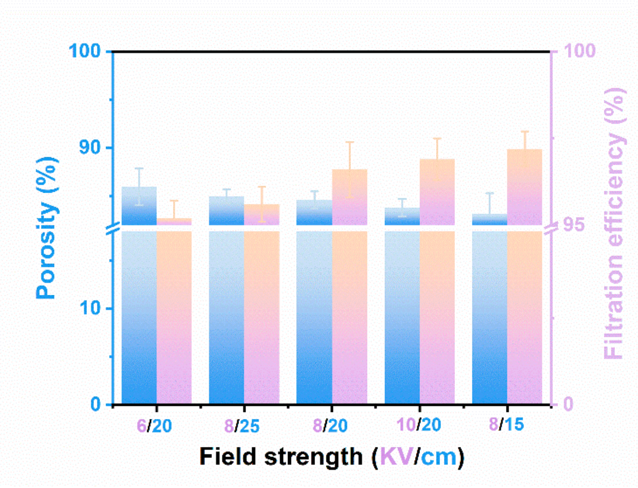

Figure S5. Effect of high voltage and tip-to-collector distance on porosity and filtration efficiency of the fiber membranes.

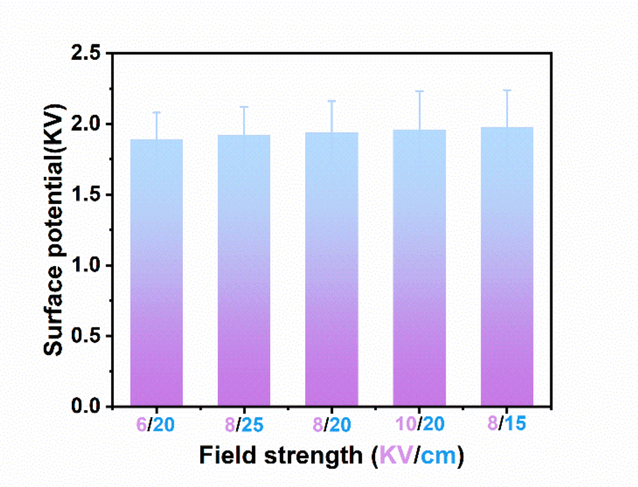

Figure S6. Effect of high voltage and tip-to-collector distance on the surface electrostatic potential of the fiber membranes.
